# Association Between Diabetic Macular Edema and Cardiovascular Events in Type 2 Diabetes Patients: A Multicenter Observational Study

**DOI:** 10.1097/MD.0000000000001220

**Published:** 2015-08-21

**Authors:** Nicolas Leveziel, Stéphanie Ragot, Elise Gand, Olivier Lichtwitz, Jean Michel Halimi, Julien Gozlan, Pierre Gourdy, Marie-Françoise Robert, Dured Dardari, Michèle Boissonnot, Ronan Roussel, Xavier Piguel, Olivier Dupuy, Florence Torremocha, Pierre-Jean Saulnier, Richard Maréchaud, Samy Hadjadj

**Affiliations:** From the Department of ophthalmology, University Hospital of Poitiers, France (NL, OL, JG, M-FR, MB); University of Poitiers, UFR Médecine et Pharmacie, France (NL, RM); U1084, Inserm, Poitiers, France (NL); Centre d’investigation clinique, University of Poitiers, Poitiers, France (SR, P-JS, SH); CIC1402, Inserm, France (SR, P-JS, SH); Centre d’investigation clinique, University Hospital of Poitiers, Poitiers, France (SR, P-JS, SH); Endocrinology and Diabetology Department, pole DUNE, University Hospital of Poitiers, Poitiers, France (EG, XP, RM, SH); Department of Nephrology-immunology, University Hospital of Tours, François Rabelais University, Tours, France (JMH); Diabetology Department, Rangueuil Hospital, University Hospital of Toulouse, France (PG); Endocrinology Department Hospital of Sud Francilien, Corbeil Essonnes, France (DD); UMRS1138, Inserm, Paris, France (RR); University Paris 7 Denis Diderot, UMRS1138, Paris, France (RR); Diabetology, endocrinology and Nutrition Department, Groupe Hospitalier Bichat Claude Bernard, Assistance Public-Hopitaux de Paris (AP-HP), Paris, France (RR); Diabetology Department Bégin Armed Forces Hospital, Saint Mandé, France (OD); U1082, Inserm, Poitiers, France (SH).

## Abstract

Diabetic macular edema (DME) is the main cause of visual loss associated with diabetes but any association between DME and cardiovascular events is unclear.

This study aims to describe the possible association between DME and cardiovascular events in a multicenter cross-sectional study of patients with type 2 diabetes.

Two thousand eight hundred seven patients with type 2 diabetes were recruited from diabetes and nephrology clinical institutional centers participating in the DIAB 2 NEPHROGENE study focusing on diabetic complications. DME (presence/absence) and diabetic retinopathy (DR) classification were based on ophthalmological report and/or on 30° color retinal photographs. DR was defined as absent, nonproliferative (background, moderate, or severe) or proliferative. Cardiovascular events were stroke, myocardial infarction, and lower limb amputation.

Details regarding associations between DME and cardiovascular events were evaluated.

The study included 2807 patients with type 2 diabetes, of whom 355 (12.6%) had DME. DME was significantly and independently associated with patient age, known duration of diabetes, HbA1c, systolic blood pressure, and DR stage. Only the prior history of lower limb amputation was strongly associated with DME in univariate and multivariate analyses, whereas no association was found with regard to myocardial infarction or stroke. Moreover, both major (n = 32) and minor lower limb (n = 96) amputations were similarly associated with DME, with respective odds ratio of 3.7 (95% confidence interval [CI], 1.77–7.74; *P* = 0.0012) and of 4.29 (95% CI, 2.79–6.61; *P* < 0.001).

DME is strongly and independently associated with lower limb amputation in type 2 diabetic patients.

## INTRODUCTION

The prevalence of diabetes is increasing worldwide, exceeding previous predictions.^1^ Patients with diabetes develop macrovascular complications that lead to a doubling of coronary deaths in addition to microvascular consequences with renal, neurological, and visual involvement.^2^ Diabetic macular edema (DME) is the main cause of visual loss associated with diabetes. Indeed, DME affects approximately 7% of diabetic patients, resulting in approximately 21 million individuals suffering from visual impairment worldwide.^3,4^ Diagnosis of clinically significant DME is important as there are several therapies associated with visual improvement such as laser grid photocoagulation and intravitreal antivascular endothelial growth factors (VEGF) injections.^5,6^

Some clinical (ie, hypertension, nephropathy) and biological parameters (ie. high glycated hemoglobin) are established risk factors for DME and cardiovascular events.^7–9^ Although previous studies have suggested that DME may be associated with cardiovascular or vascular events,^10–12^ the relationship between DME and the general cardiovascular consequences of diabetes including lower limb amputation requires further investigations. Particularly because none of the studies linking diabetic retinopathy (DR) with cardiovascular events separated ischemic retinal lesions from DME.^13^

The objective of the present study was to describe the association between retinal involvement and cardiovascular events in a multicenter study of type 2 diabetic patients, with special focus on DME and DR.

## METHODS

### Patient Recruitment

Type 2 diabetic patients were recruited in diabetes and nephrology clinical centers participating in the DIAB 2 NEPHROGENE study (see list in appendix). The aim of this French multicenter cross-sectional study was to explore the contribution of genes and environment to type 2 diabetes complication phenotypes. This ancillary study focuses on clinical or biological associations with DME.

Selection criteria have been previously described^14^ and comprised patients with type 2 diabetes with or without DME. The study design was approved by the local ethics committee (CPP Ouest III) and all participants gave written informed consent.

### Definition of Cardiovascular Endpoints

Any history of prior cardiovascular events—myocardial infarction, stroke and lower limb amputation—was recorded at inclusion, from the patient record. At inclusion of patients, an electrocardiogram (ECG) was performed to assess any previous myocardial infarction. In cases of multiple lower limb amputations, the most severe level was considered: transtibial or transfemoral amputations were classified as major whereas transmetatarsal or toe amputations were classified as minor amputations.

### Ophthalmological Classification

Diagnosis of DME was based on 30° nonmydriatic retinal color photographs interpreted by a senior ophthalmologist and/or on fundus examination after pupillary dilatation performed by a senior ophthalmologist.

Diagnosis of DME was defined as a localized or diffuse thickening of the macular area usually associated with retinal exudates, cysts, and microanevrisms. Patients who could not be assessed for DME, for poor quality of retinal photographs or failure to attend ophthalmological examination during the study, were not considered for analysis in the present study.

DR was defined as absent, nonproliferative (background, moderate, or severe) or proliferative based on 30° retinal color photographs covering the 7 fields and graded according to the Modified Airlie House final classification. In this analysis, 2 groups of patients were classified according to the Early Treatment Diabetic Retinopathy Study (ETDRS) classification^15^: those graded less than stage 53 (group 1) and those graded stage 53 or more (group 2).

Patients were classified according to the eye with the more severe stage.

### Biological Determination

Serum creatinine and urinary albumin were measured by nephelometry on a Modular System P (Roche Diagnostics GmbH, Penzberg, Germany). Renal function was estimated glomerular filtration rate (eGFR) using the Modification of Diet in Renal disease (MDRD-4) formula. Urinary creatinine was measured on a Hitachi911 automatic analyzer (Roche Diagnostics, Meylan, France). Glycated hemoglobin (A1C) was determined by using a high performance liquid chromatography method with an ADAMS A1C HA-8160 analyzer (normal values 4.0% to 6.0%; Menarini, Florence, Italy).

### Statistical Analyses

Statistical analyses were performed with Statview 5.0 (SAS Institute, Cary, NC). Patients’ characteristics are expressed as means ± standard deviation or medians (interquartile range) for skewed distributions.

Intergroup comparisons were performed using logistic regression for both univariate and multivariate approaches. Results are given as odds ratio (OR) and 95% confidence intervals (CIs).

Stratification was performed on the presence of significant DR (group 1 vs group 2), due to a significant interaction (*P* < 0.001) between lower limb amputation and DR for the relation between DME and lower limb amputation.

Multivariate logistic regression analyses with DME as the dependent variable were performed using a backward manual procedure; variables with *P* < 0.05 in univariate analysis were selected in the maximal model.

All comparisons were 2 sided with *P* < 0.05 being considered statistically significant.

## RESULTS

### Demographic Data

The DIAB 2 NEPHROGENE study included 2807 patients with type 2 diabetes after exclusion of patients due to uncertainty about the presence or absence of DME. This study population consisted in 355 patients (12.6%) with DME and 2452 patients (87.4%) without DME. When considering the whole population, history of lower limb amputation was reported in 82/2370 (3.46%) among patient without DME and in 46/309 (12.96%) among patients with DME (*P* < 0.001).

History of stroke and myocardial infarction was reported in 111/2341 (4.53%) and 306/2146 (12.48%) among patients without DME and in 27/328 (7.61%) and 52/303 (14.65%), respectively, among patients with DME (*P* = 0.01 and 0.25, respectively). Their general characteristics are detailed in Table 1.

**TABLE 1:**
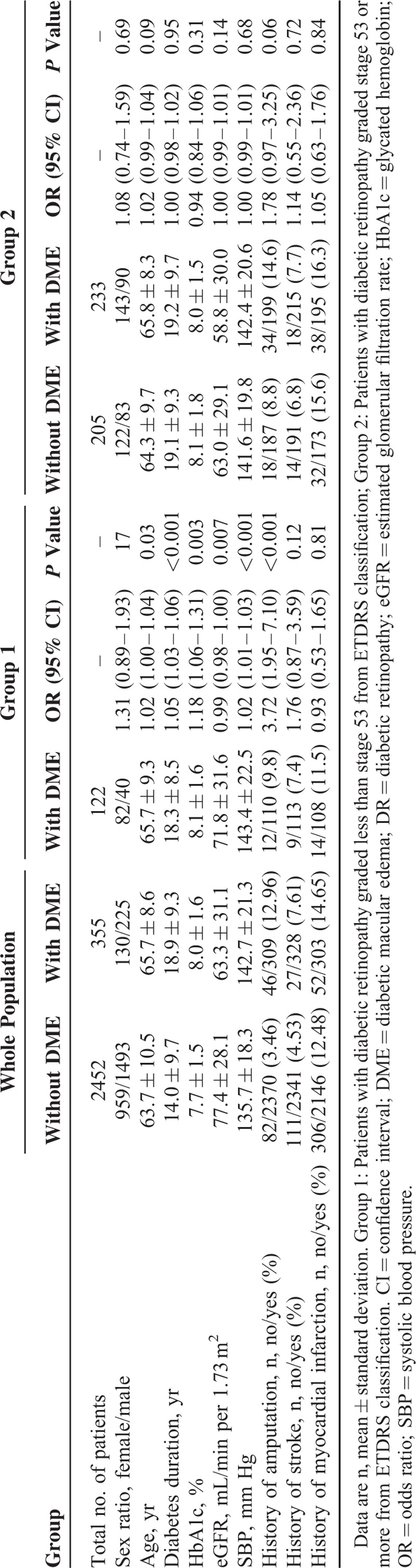
Comparison of Patients With and Without DME, With Focus on DR Severity Stage: Univariate Analyses for Demographic, Clinical, and Biological Variables

### DME, DR, and Cardiovascular Events—Univariate Analysis

In the study population, DME was significantly associated with age, known duration of diabetes, HbA1c eGFR, and systolic blood pressure (SBP) (Table 1). As expected, DME was significantly more frequent as DR stage became more severe (χ^2^ for trend: 696, *P* < 0.001) (see details in Table 2).

**TABLE 2:**
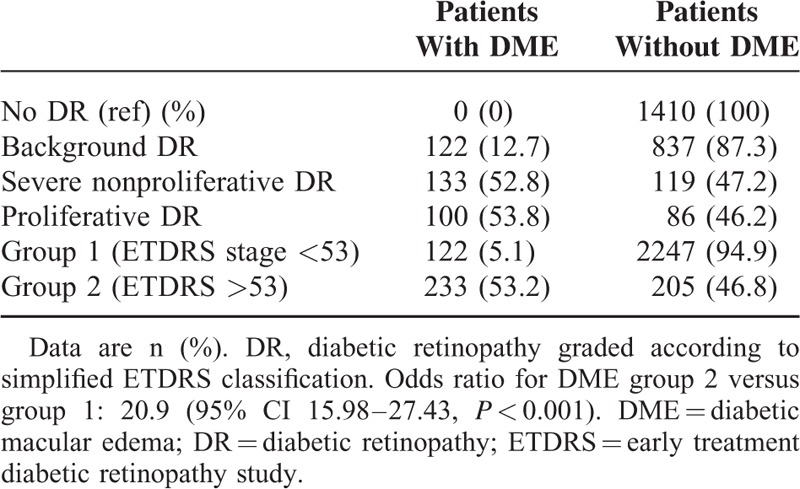
Association Between DME and Diabetic Retinopathy

In group 1 (patients with DR graded less than stage 53), DME was significantly associated with age, known duration of diabetes, HbA1c, eGFR, SBP, and with a prior history of lower limb amputation (Table 1).

None of the previous clinical or biological variables remained associated with DME when sub-analysis was carried out on group 2 (patients graded stage 53 or more).

We investigated the relationship between DME and DR. In this context, DME was associated with DR. These data are presented in Table 2. We also investigated the relationship between the level of lower limb amputation and DME. No trend was found for an association between DME and severity of lower limb amputation. Indeed, both major and minor lower limb amputations were similarly associated with DME, with respective OR of 3.7 (95% CI, 1.77–7.74; *P* < 0.0012) and of 4.29 (95% CI, 2.79–6.61; *P* < 0.001) (Table 3).

**TABLE 3:**
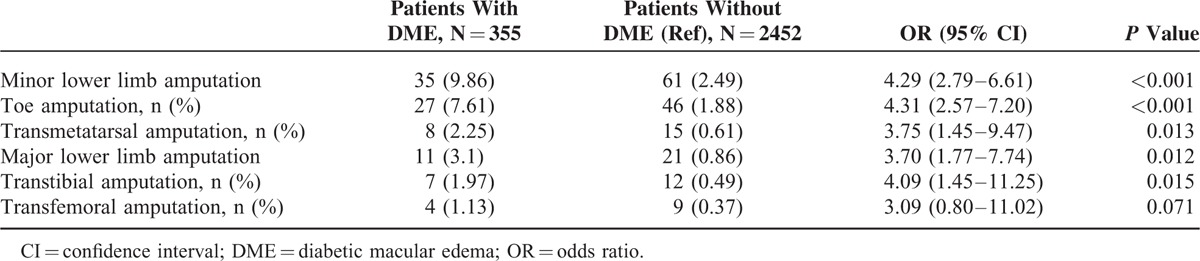
Association Between DME and Severity of Lower Limb Amputation—Univariate Analysis

In the present study, DR was associated with cardiovascular events (Table 4). In fact, when compared to patients with no DR, DR was associated with SBP (*P* < 0.001), lower limb amputation (*P* < 0.001), stroke (*P* = 0.0051), and myocardial infarction (*P* = 0.035).

**TABLE 4:**
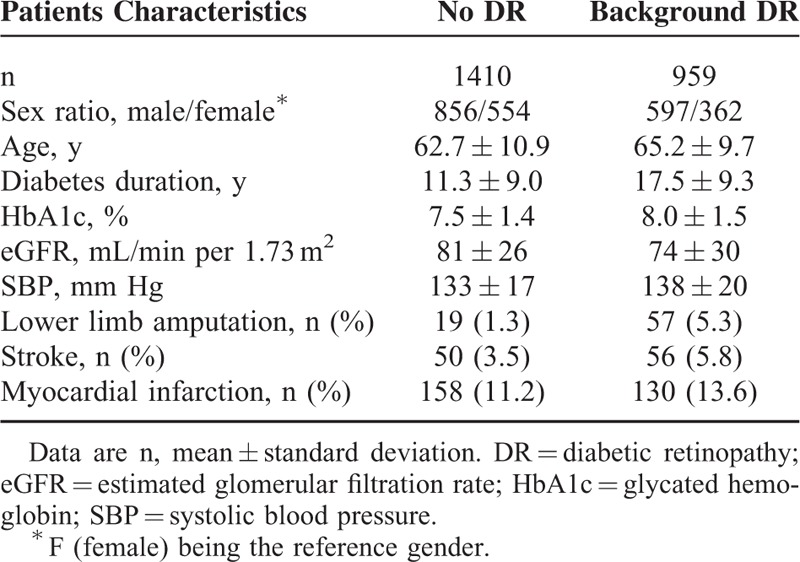
Univariate Analyses for Cardiovascular Events and Biological Variables Associated to Nonedematous Diabetic Retinopathy

### DME and Cardiovascular Events—Multivariate Analysis

Multivariate analyses carried out on the study population showed that the risk factors identified in univariate analysis remained independently associated with DME. Indeed, as previously observed in univariate analyses, significant associations persisted between lower limb amputation and DME in the whole cohort and in group 1 (Tables 5 and 6), whereas no association was found in group 2 (data not shown).

**TABLE 5:**
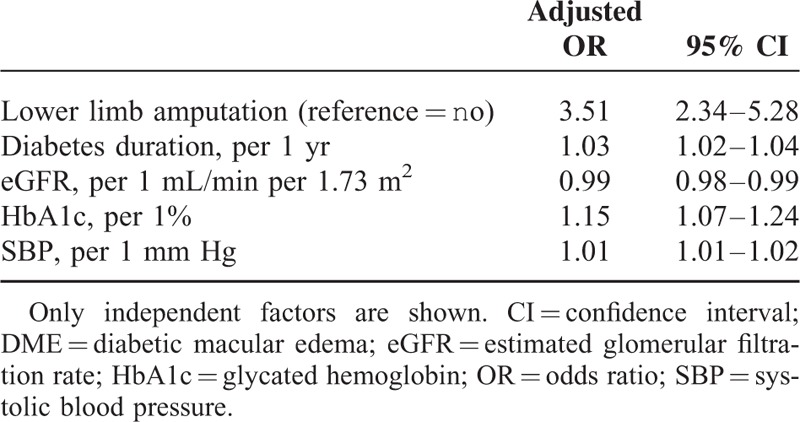
Multivariate Logistic Regression of DME in the Study Population

**TABLE 6:**
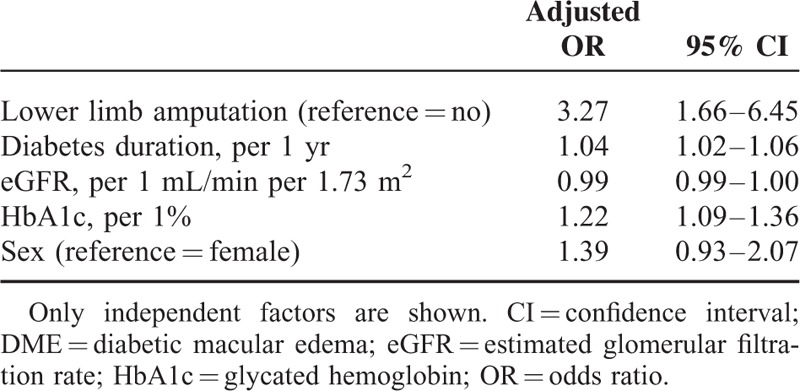
Multivariate Logistic Regression of DME in Group 1

Interestingly, after full adjustment, history of lower limb amputation remained more than doubled in cases of DME.

## DISCUSSION

### Association With Cardiovascular Events

In this large-scale cross-sectional analysis, we found an association between cardiovascular risk factors such as age and SBP, and DME. In addition, DME was significantly associated with lower limb amputation in type 2 diabetes patients. In multivariate analysis, SBP and prior history of lower limb amputation were positively and significantly associated with DME. This association between DME and lower limb amputation was independent of DR. The present study did not establish any significant association between myocardial infarction and DME either in our study population or in subgroup analyses. Furthermore, an association was observed between stroke and DME only in univariate analysis performed in the whole population.

A recent retrospective study comparing incidence rates of myocardial infarction and cerebrovascular accident requiring hospitalization in DME patients and in diabetic patients without retinal involvement, found significantly more cardiovascular events, such as myocardial infarction and cerebrovascular events, in patients with DME.^10^ Our study did not confirm the hypothesized link between myocardial infarction or stroke and DME. This discrepancy may be explained by differences between the 2 populations in terms of age at inclusion and cardiovascular events. DME had previously been associated with lower limb amputation in the Veterans Affairs Diabetes Trial, a study including 1268 patients with type 2 diabetes with different ethnicities, whereas no association could be established with regard to stroke or myocardial infarction in agreement with our findings.^16^ In the Veterans Affairs Diabetes Trial study, 7.9% of patients with DME (n = 127) versus 1.8% of patients without DME (n = 1141) had a history of lower limb amputation. Here we report markedly higher rates of lower limb amputation, which was undergone by 12.96% of patients with DME and by 3.8% of patients without DME. These differences may also be explained by differences in the characteristics of patients recruited in the 2 cohorts.

Associations have also been established in our cohort between DR and cardiovascular events. Lower limb amputation was associated with all DR stages, whereas stroke and myocardial infarction were associated primarily with proliferative DR. Previous studies had investigated the presence of DR as a predictor of cardiovascular morbidity and mortality^17–22^ and a recent meta-analysis of 20 observational studies established significant associations between DR and all-cause mortality and/or cardiovascular events (such as myocardial infarction, angina pectoris, coronary artery bypass graft, ischemic changes on ECG, transient ischemic attack, nonfatal stroke, or lower limb amputation).^11^ However, the study lacks any further information on DME and does not inform on the relationship between DR and lower limb amputation.

As we analyzed the relationship between DME and lower limb amputation, we found that while a relationship between cardiovascular events and DME existed in our study population taken as a whole and in group 1 (patients graded less than stage 53), no such association could be established in group 2 (patients graded stage 53 or more). This is probably due to the strong association existing between DME and DR in this subgroup, which most likely overwhelmed any other potential associations with minor effects. Regarding the clinical criteria of lower limb amputation, all degrees of lower limb amputation were taken into account in our study, without consideration of the pathophysiological mechanisms involved that is peripheral neuropathy, peripheral artery disease, or infection.^23^ Interestingly, a sub-analysis taking into account the level of lower limb amputation suggested that DME was similarly associated with both microvascular and macrovascular processes, while a more strongly pronounced relationship between microvascular disease, that is “minor amputations” and DME was intuitively expected.

In the Field Study including 9795 type 2 diabetic patients randomized to fenofibrate or placebo, lower limb amputation was more significantly associated with DR than with myocardial infarctions or strokes. Among patients with minor amputations, 36% had DR while among patients with major amputations, only 26% suffered from this condition.^24^ Our data corroborated these results by underlining the extent to which lower limb amputation is more closely associated with DR than with stroke or myocardial infarction.

One key question arising from the analysis of our data is whether any single unifying mechanism interlinks DR, DME, and lower limb amputation. Biological mechanisms that lead to DME or to DR share many similarities but are also markedly different. DME is produced by alterations of tight junctions, pericyte and endothelial cell loss, retinal vessel leukostasis and increased permeability of retinal capillaries and retinal pigment epithelium cells.^25^ On the other hand, the ischemic territories formed by retinal capillary occlusions leading to DR are caused mainly by wall modifications through thickening of basement membranes, pericytes, and endothelial cell loss combined with leukostasis. Extension of the ischemic territories leads to heightened expression of the growth factors (VEGF, IGF-1) that are involved in the retinal neovascular process.^26–28^ To explain the interaction between lower limb amputation and severe DR and DME, we can speculate that the occlusive microvascular processes observed in the retina are likely to be stronger determinants than the exudative processes due to VEGF upregulation. The capillary occlusions underlying ischemic DR may also to some extent reflect the peripheral artery disease leading to lower limb amputation. Capillary occlusion might also be involved in the development of diabetic neuropathy, another important contributor to lower limb amputation. On the other hand, a different mechanism entirely could help to explain the association between DME and lower limb amputation. Several disorders associated with central visual impairment that have been shown to increase the risk of fall and traumatic injuries,^29^ and it is possible that the visual impairment caused by DME itself may contribute to foot ulcer.

### Study Limitations

We acknowledge some limitations regarding the study methodology we used for DME diagnosis, which was based on nonmydriatic color photographs interpreted by senior ophthalmologists. In this context, it is likely that clinically nonsignificant DME with no exudates and without significant visual acuity decrease remained undetected. However, other studies have demonstrated close agreement with regard to DME severity levels (κ = 0.92)^30^ and to DME detection (κ = 0.97)^31^ between nonmydriatic color photographs and the gold standard reference of color stereo-photographs of 7 standard fields. Similarly, a 2001 systematic review concluded that single-field fundus photographs for screening of DR have sensitivity ranging from 61% to 90% and specificity ranging from 85% to 97% when compared with the stereo-photographs of 7 standard fields.^32^

All of these methods for DME diagnosis rely on en face determination of retinal thickening, and a recent study suggested that color photographs could be a less reliable tool, when compared to optical coherence tomography as a means of detecting DME.^33^ However, while this method of DME determination is widely used in interventional clinical trials to assess the effect of molecules on DME during the treatment, it is not currently considered as the gold standard for DME determination in diabetic screening, likely due to the fact that OCT equipment is not as widely used in screening centers as color retinographs. Our study design is cross-sectional, which renders it impossible to establish a causal relationship between DME and lower limb amputation. We were also unable to establish any temporal relationship between visual impairment and lower limb amputation and it is possible that the association observed was produced by recruitment bias as the centers included in the study specialize in diabetes and nephropathy and may have a skewed population of patients with diabetes and severe kidney disease. Our method for establishing the presence of DME was also suboptimal and may have underreported DME as retinal photographs may not pick up DME in the absence of exudates. All of the images and patients were graded by the same clinician but we did not assess the repeatability of those assessments of the diabetes photographs. Four percent of our sample was excluded due to inadequate characterization of the macular status which may have skewed our results. Our method of ascertainment also relied on reported history. Cardiovascular outcomes and stroke while sometimes obvious are less easily characterized than lower limb amputation and these may have been underreported. Nevertheless, we had a large sample size and a cross-sectional approach has high statistical power through which relatively rare events such as amputation can be taken into adequate account.

Our general conclusion following this large-scale study is that DME and proliferative DR are strong and independent risk factors for lower limb amputation. Indeed, the main finding is that in this study people with macular edema had a 3.5-fold increased risk for lower limb amputation. In the context of DR screening, assessment of both retinopathy and macular status may help to identify patients at high risk for amputation. Equally patients with a prior history of amputation should be given special attention. A prospective approach may clarify the prognostic value of DME in relation to the risk of minor or major lower limb amputation in type 2 diabetes patients.

## References

[1] Zimmet P Alberti KG Shaw J Global and societal implications of the diabetes epidemic. *Nature* 2001; 414:782–787. 11742409 10.1038/414782a

[2] Haffner SM Lehto S Rönnemaa T Mortality from coronary heart disease in subjects with type 2 diabetes and in nondiabetic subjects with and without prior myocardial infarction. *N Engl J Med* 1998; 339:229–234. 9673301 10.1056/NEJM199807233390404

[3] Ding J Wong TY Current epidemiology of diabetic retinopathy and diabetic macular edema. *Curr Diab Rep* 2012; 12:346–354. 22585044 10.1007/s11892-012-0283-6

[4] Romero-Aroca P Managing diabetic macular edema: the leading cause of diabetes blindness. *World J Diabetes* 2011; 2:98–104. 21860693 10.4239/wjd.v2.i6.98 PMC3158878

[5] Photocoagulation for diabetic macular edema. Early Treatment Diabetic Retinopathy Study report number 1. Early Treatment Diabetic Retinopathy Study research group. *Arch Ophthalmol* 1985; 103:1796–1806. 2866759

[6] Chun DW Heier JS Topping TM A pilot study of multiple intravitreal injections of ranibizumab in patients with center-involving clinically significant diabetic macular edema. *Ophthalmology* 2006; 113:1706–1712. 17011952 10.1016/j.ophtha.2006.04.033

[7] Stratton IM Kohner EM Aldington SJ UKPDS 50: risk factors for incidence and progression of retinopathy in Type II diabetes over 6 years from diagnosis. *Diabetologica* 2001; 44:156–163. 10.1007/s001250051594 11270671

[8] Klein R Klein BE Moss SE The Wisconsin Epidemiologic Study of Diabetic Retinopathy. XV. The long term incidence of macular edema. *Ophthalmology* 1995; 102:7–16. 7831044 10.1016/s0161-6420(95)31052-4

[9] Klein R Knudtson MD Lee KE The Wisconsin Epidemiologic Study of Diabetic Retinopathy XXIII: the twenty-five-year incidence of macular edema in persons with type 1 diabetes. *Ophthalmology* 2009; 116:497–503. 19167079 10.1016/j.ophtha.2008.10.016 PMC2693093

[10] Nguyen-Khoa BA Goehring EL Jr Werther W Hospitalized cardiovascular events in patients with diabetic macular edema. *BMC Ophthalmol* 2012; 12:11. 22646811 10.1186/1471-2415-12-11 PMC3395554

[11] Kramer CK Rodrigues TC Canani LH Diabetic retinopathy predicts all-cause mortality and cardiovascular events in both type 1 and 2 diabetes: meta-analysis of observational studies. *Diabetes Care* 2011; 34:1238–1244. 21525504 10.2337/dc11-0079 PMC3114518

[12] Kawasaki R Cheung N Islam FM Is diabetic retinopathy related to subclinical cardiovascular disease? *Ophthalmology* 2011; 118:860–865. 21168222 10.1016/j.ophtha.2010.08.040 PMC3087839

[13] Penno G Solini A Bonora E HbA1c variability as an independent correlate of nephropathy, but not retinopathy, in patients with type 2 diabetes: The Renal Insufficiency And Cardiovascular Events (RIACE) Italian multicenter study. *Diabetes Care* 2013; 36:2301–2310. 23491522 10.2337/dc12-2264 PMC3714498

[14] Saulnier PJ Roussel R Halimi JM Impact of natriuretic peptide clearance receptor (NPR3) gene variants on blood pressure in type 2 diabetes. *Diabetes Care* 2011; 34:1199–1204. 21464461 10.2337/dc10-2057 PMC3114497

[15] Early Treatment Diabetic Retinopathy Study Research Group. Grading diabetic retinopathy from stereoscopic color fundus photographs. An extension of the modified Airlie House Classification. ETDRS report number 10. *Ophthalmology* 1991; 98:786–806. 2062513

[16] Emanuele N Moritz T Klein R Ethnicity, race, and clinically significant macular edema in the Veterans Affairs Diabetes Trial (VADT). *Diabetes Res Clin Pract* 2009; 86:104–110. 19720420 10.1016/j.diabres.2009.08.001

[17] Miettinen H Haffner SM Lehto S Retinopathy predicts coronary heart disease events in NIDDM patients. *Diabetes Care* 1996; 19:1445–1448. 8941482 10.2337/diacare.19.12.1445

[18] Van Hecke MV Dekker JM Stehouwer CD Diabetic retinopathy is associated with mortality and cardiovascular disease incidence: the EURODIAB prospective complications study. *Diabetes Care* 2005; 28:1383–1389. 15920056 10.2337/diacare.28.6.1383

[19] Cheung N Rogers S Couper DJ Is diabetic retinopathy an independent risk factor for ischemic stroke? *Stroke* 2007; 38:398–401. 17194880 10.1161/01.STR.0000254547.91276.50

[20] Targher G Bertolini L Zenari L Diabetic retinopathy is associated with an increased incidence of cardiovascular events in Type 2 diabetic patients. *Diabet Med* 2008; 25:45–50. 18199131 10.1111/j.1464-5491.2007.02327.x

[21] Liew G Wong TY Mitchell P Retinopathy predicts coronary heart disease mortality. *Heart* 2009; 95:391–394. 18697802 10.1136/hrt.2008.146670

[22] Klein R Klein BE Moss SE Association of ocular disease and mortality in a diabetic population. *Arch Ophthalmol* 1999; 117:1487–1495. 10565517 10.1001/archopht.117.11.1487

[23] Cheer K Shearman C Jude EB Managing complications of the diabetic foot. *BMJ* 2009; 339:b4905. 19955124 10.1136/bmj.b4905

[24] Rajamani K Colman PG Li LP Effect of fenofibrate on amputation events in people with type 2 diabetes mellitus (FIELD study): a prespecified analysis of a randomised controlled trial. *Lancet* 2009; 373:1780–1788. 19465233 10.1016/S0140-6736(09)60698-X PMC2687887

[25] Romero-Aroca P Targeting the pathophysiology of diabetic macular edema. *Diabetes Care* 2010; 33:2484–2485. 20980428 10.2337/dc10-1580 PMC2963519

[26] Grant M Russell B Fitzgerald C Insulin-like growth factor in vitreous. Studies in control and diabetic subjects with neovascularization. *Diabetes* 1986; 35:416–420. 2420665 10.2337/diab.35.4.416

[27] Meyer-Schwickerath R Pfeiffer A Blum WF Vitreous levels of the insulin-like growth factors I and II, and the insulin-like growth factor binding protein 2 and 3, increase in neovascular eye disease. Studies in nondiabetic and diabetic subjects. *J Clin Invest* 1993; 92:2620–2625. 7504689 10.1172/JCI116877 PMC288458

[28] Adamis AP Miller JW Bernal MT Increased vascular endothelial growth factor levels in the vitreous of eyes with proliferative diabetic retinopathy. *Am J Ophthalmol* 1994; 118:445–450. 7943121 10.1016/s0002-9394(14)75794-0

[29] Wood JM Lacherez P Black AA Risk of falls, injurious falls, and other injuries resulting from visual impairment among older adults with age-related macular degeneration. *Invest Ophthalmol Vis Sci* 2011; 52:5088–5092. 21474773 10.1167/iovs.10-6644

[30] Silva PS Walia S Cavallerano JD Comparison of low-light nonmydriatic digital imaging with 35-mm ETDRS seven-standard field stereo color fundus photographs and clinical examination. *Telemed J E Health* 2012; 18:492–499. 22827402 10.1089/tmj.2011.0232

[31] Vujosevic S Benetti E Massignan F Screening for diabetic retinopathy: 1 and 3 nonmydriatic 45-degree digital fundus photographs vs 7 standard early treatment diabetic retinopathy study fields. *Am J Ophthalmol* 2009; 148:111–118. 19406376 10.1016/j.ajo.2009.02.031

[32] Williams GA Scott IU Haller JA Single-field fundus photography for diabetic retinopathy screening: a report by the American Academy of Ophthalmology. *Ophthalmology* 2004; 111:1055–1062. 15121388 10.1016/j.ophtha.2004.02.004

[33] Mackenzie S Schmermer C Charnley A SDOCT imaging to identify macular pathology in patients diagnosed with diabetic maculopathy by a digital photographic retinal screening programme. *PLoS ONE* 2011; 6:e14811. 21573106 10.1371/journal.pone.0014811 PMC3089611

